# Security-Aware Codebook Design for Low-PAPR AFDM Systems

**DOI:** 10.3390/s26113614

**Published:** 2026-06-05

**Authors:** Tingting Zhang, Haibo Dai

**Affiliations:** 1 School of Communication and Information Engineering, Nanjing University of Posts and Telecommunications, Nanjing 210023, China; 2022010309@njupt.edu.cn; 2 School of Internet of Things, Nanjing University of Posts and Telecommunications, Nanjing 210023, China

**Keywords:** affine frequency-division multiplexing, peak-to-average power ratio, physical-layer security, blind side-information detection, codebook design

## Abstract

Affine frequency division multiplexing (AFDM) is regarded as a promising waveform for high-mobility wireless systems. However, the public codebook used in AFDM raises security concerns when the link is observed by an eavesdropper, and meanwhile AFDM communication suffers from a high peak-to-average power ratio (PAPR). This paper proposes a security-aware codebook design for low-PAPR AFDM systems. Specifically, the codebook is designed to minimize an eavesdropper-oriented cross-alignment metric while maintaining the legitimate user’s decoding reliability and keeping the PAPR low. Since the resulting design problem is non-convex, we develop a dedicated alternating discrete coordinate descent algorithm to solve it. Simulation results show that the proposed codebook design significantly degrades the eavesdropper’s decoding performance without degrading that of the legitimate receiver while maintaining the low-PAPR.

## 1. Introduction

Future high-mobility wireless systems operate over doubly dispersive channels, where delay and Doppler spreads appear simultaneously and severely degrade the performance of conventional orthogonal multicarrier schemes such as orthogonal frequency division multiplexing (OFDM). To address this challenge, affine frequency division multiplexing (AFDM) has recently been proposed as a waveform built on the discrete affine Fourier transform [1,2]. In AFDM, two scalar chirp parameters determine the modulation kernel, and data symbols are mapped onto quadratic-phase subcarriers that yield a structured channel representation in the affine domain. With proper parameter design, AFDM achieves full diversity and remains robust in rapidly time-varying channels [3]. The same structure also benefits sensing, since the delay–Doppler representation of the channel supports target parameter extraction and is closely related to the ambiguity function used in range and velocity estimation [4,5]. These properties make AFDM attractive for high-mobility applications such as high-speed rail communications, vehicular links, and integrated sensing and communications.

These properties have motivated extensive research on AFDM. For integrated sensing and communications (ISAC), the authors in [6] investigate sensing with AFDM pilots and develop the angle–delay–Doppler estimation for mixed near-field and far-field scenarios. On the receiver and transceiver side, channel estimation for multiple-input multiple-output (MIMO) AFDM and efficient precoding for extremely large-scale MIMO AFDM are studied in [7,8], respectively. AFDM has also been combined with other transmission techniques, including index modulation, pre-chirp-domain index modulation, and AFDM-SCMA for massive connectivity over high-mobility channels [9,10,11]. In addition, reliability under hostile interference is addressed in [12], and chirp permutation is exploited in [5] to enlarge the design space for sensing-aware transmission.

Among the practical issues of AFDM, peak-to-average power ratio (PAPR) reduction has received particular attention, since a large signal envelope drives the power amplifier into nonlinear operation and degrades the bit error rate [13]. Along this line, ref. [14] proposes a grouped pre-chirp selection algorithm that varies the pre-chirp parameter across subcarrier groups and selects the candidate with the lowest PAPR. A premodulation data spreading framework based on well-established transforms is developed in [15] and can reduce the PAPR without side-information overhead. In [16], a deep neural network replaces the guard intervals of pilot-embedded AFDM signals with low-amplitude symbols and performs active constellation extension to reduce the PAPR. More recently [17], introduces per-slot scalar chirp-offset selection with a small codebook that enables blind side-information detection at the receiver without explicit signaling. A related waveform-level effort is reported in [18], where affine filter bank modulation is developed to further reduce PAPR and out-of-band emission. In parallel, physical-layer security has become an increasingly important concern for open high-mobility links. In this direction, secure AFDM design for high-mobility environments, chirp-parameter hopping, secure waveform design for satellite–air integrated communications, and robustness against passive eavesdroppers are investigated in [19,20,21,22], and a time-varying secure AFDM design is reported in [23]. However, these two lines of work have remained largely separate, and the joint design of secure and low-PAPR AFDM is still an open problem.

To fill this gap, this paper proposes a security-aware codebook design for low-PAPR AFDM systems. We consider an AFDM system consisting of a transmitter, a legitimate receiver, and an eavesdropper, where the transmitter and the legitimate receiver share a secret scalar offset on the second chirp parameter. Under this setup, we formulate the codebook design as an optimization problem that minimizes an eavesdropper-oriented cross-alignment metric subject to a legitimate-user separability constraint and a PAPR-budget constraint. To make the problem tractable, the codebook is restricted to a structured integer-grid family, which yields a non-convex discrete integer program. We then develop a dedicated alternating discrete coordinate descent algorithm to solve it. Simulation results show that the proposed algorithm significantly increases the eavesdropper’s BER without increasing the legitimate receiver’s BER while keeping the low PAPR.

The main contributions of this paper are summarized as follows:

- To the best of our knowledge, this is the first work that jointly addresses security and PAPR reduction in AFDM systems. Specifically, we propose a security-aware codebook design for low-PAPR AFDM systems, which minimizes an eavesdropper-oriented cross-alignment metric subject to a legitimate-user separability constraint and a PAPR-budget constraint.
- To make the problem tractable, we restrict the codebook to a structured integer-grid family, which yields a non-convex discrete integer program. We then develop a dedicated alternating discrete coordinate descent algorithm to solve it, in which the integer coordinates are updated cyclically and each coordinate is optimized by an exact one-dimensional search over its feasible range while the remaining coordinates are fixed.
- Simulation results show that the proposed codebook design significantly increases the eavesdropper’s BER without increasing the legitimate receiver’s BER while keeping the low PAPR, and attains the performance of the exhaustive-search benchmark. Meanwhile, the results demonstrate the trade-off between the PAPR budget and the performance of secure communication.

The remainder of this paper is organized as follows. Section 2 presents the secure AFDM signal and system models. Section 3 formulates the security-aware codebook design problem and describes the alternating discrete coordinate descent algorithm. Section 4 provides simulation results, and Section 5 concludes the paper.

Notation: Boldface lower-case and upper-case letters denote vectors and matrices, respectively. ${\left(\cdot \right)}^{H}$ denotes the Hermitian transpose. C denotes the complex field. E{·} stands for expectation. The operator Q(·) denotes the nearest-neighbor slicer.

## 2. Signal and System Models

In this section, we first introduce the transmitted signal model for low-PAPR AFDM with the secret offset in Section 2.1, and then describe the receiver models for the legitimate user and the eavesdropper in Section 2.2.

### 2.1. Transmitted Signal Model with Secret Offset

We consider an AFDM system with one transmitter (Alice), one legitimate receiver (Bob), and one eavesdropper (Eve). Alice transmits confidential symbols to Bob over a doubly dispersive channel, while Eve passively observes the transmission and attempts to decode the same symbols.

Following the frame structure in [17], each transmission consists of $N_{\mathrm{sl}}$ slots, and each slot carries a length-*M* data symbol vector $x={\left[x\left[0\right],\ldots ,x\left[M-1\right]\right]}^{T}\in C^{M}$ of unit average power, i.e., $E[|x\left[m\right]{|}^{2}]=1$. The AFDM modulation is characterized by the discrete affine Fourier transform (DAFT) matrix $E\left(c_{1},c_{2}\right)\in C^{M\times M}$, whose (n,m)-th entry is

(1) $${\left[E(c_{1},c_{2})\right]}_{n,m}=\frac{1}{\sqrt{M}}\exp\;\left(j2\pi \left(c_{1}n^{2}+c_{2}m^{2}+\frac{nm}{M}\right)\right),$$

for n,m=0,…,M−1, with two scalar chirp parameters $c_{1}$ and $c_{2}$.

As in [17], $c_{1}$ is fixed across slots, while $c_{2}$ is selected per slot from a codebook of *U* candidates indexed by u=1,…,U. To introduce security, we let the second chirp parameter under candidate *u* take the form

(2) $$c_{2}^{\left(u\right)}=\kappa_{AB}+\delta^{\left(u\right)},\;u=1,\ldots ,U,$$

where $\kappa_{AB}$ is a shared secret offset known only to Alice and Bob, and $\delta^{\left(u\right)}$ is a public relative offset drawn from the relative codebook

(3) $$D=\left\{\delta^{\left(1\right)},\delta^{\left(2\right)},\ldots ,\delta^{\left(U\right)}\right\}$$

that is accessible to every receiver including Eve. The secret offset $\kappa_{AB}$ creates a de-chirping mismatch at any receiver that does not know it, whereas the public offsets $\left\{\delta^{\left(u\right)}\right\}$ retain the candidate diversity used for per-slot PAPR reduction and blind side-information detection.

Under candidate *u*, the symbol-rate transmitted waveform is then

(4) $$s_{u}=E\left(c_{1},c_{2}^{\left(u\right)}\right)x.$$

Since PAPR is defined on the continuous-time waveform, a ζ-times denser time grid is needed for a reliable empirical estimate. Replacing the integer time index *n* in (1) with the fractional index $\tilde{n}=n/\zeta$ yields the ζ-times oversampled waveform

(5) $${\bar{s}}_{u}\left[n\right]=\frac{1}{\sqrt{M}}\sum_{m=0}^{M-1}x\left[m\right]\exp\;\left(j2\pi \left(c_{1}{\tilde{n}}^{\;2}+c_{2}^{\left(u\right)}m^{2}+\frac{\tilde{n}m}{M}\right)\right),$$

where ζ≥1 is the oversampling factor, n=0,…,ζM−1, and ${\bar{s}}_{u}\left[k\zeta \right]={\left[s_{u}\right]}_{k}$ at the symbol-rate indices k=0,…,M−1.

For each slot, the transmitter selects the candidate that yields the lowest PAPR, i.e.,

(6) $$u^{★}=\arg\min_{1\leq u\leq U}\mathrm{PAPR}\left(s_{u}\right),$$

where $\mathrm{PAPR}\left(s_{u}\right)≜\max_{n}|{\bar{s}}_{u}\left[n\right]{|}^{2}/E\{|{\bar{s}}_{u}\left[n\right]{|}^{2}\}$ is evaluated on the oversampled waveform ${\bar{s}}_{u}\left[n\right]$.

### 2.2. Receiver and Blind Side-Information Detection

The transmitted waveform propagates to Bob and Eve over independent doubly dispersive channels and is corrupted by additive noise at each receiver. After matched filtering and symbol-rate sampling, the received signals at Bob and Eve are modeled as

(7) $$r_{i}=\sqrt{P_{\mathrm{in}}}\;H_{i}s_{u^{★}}+w_{i},\;i\in \left\{B,E\right\},$$

where $P_{\mathrm{in}}$ is the average symbol-rate transmit power, $H_{i}\in C^{M\times M}$ is the effective channel matrix observed by receiver *i*, and $w_{i}∼\mathrm{CN}\left(0,N_{0}I\right)$ is the received noise.

Each receiver applies a linear minimum mean-square error (LMMSE) equalizer to estimate the transmitted modulated waveform $s_{u^{★}}$ as

(8) $${\hat{s}}_{i}={\left(P_{\mathrm{in}}H_{i}^{H}H_{i}+N_{0}I\right)}^{-1}\sqrt{P_{\mathrm{in}}}\;H_{i}^{H}r_{i},\;i\in \left\{B,E\right\},$$

which is independent of the candidate index and hence computed once per slot. AFDM demodulation further requires the second chirp parameter $c_{2}$ used at the transmitter, on which Bob and Eve have different information.

Bob knows the secret offset $\kappa_{AB}$ and can therefore enumerate all *U* candidates ${\left\{c_{2}^{\left(u\right)}\right\}}_{u=1}^{U}$ in (2), although the specific candidate $c_{2}^{\left(u^{★}\right)}$ used in a given slot is not signaled to him. Bob therefore estimates $u^{★}$ blindly by testing every candidate through the unitary projection

(9) $${\hat{x}}_{u}^{\left(B\right)}=E^{H}\left(c_{1},\kappa_{AB}+\delta^{\left(u\right)}\right)\;{\hat{s}}_{B},\;u=1,\ldots ,U.$$

Because $E(c_{1},c_{2})$ is unitary, $u=u^{★}$ cancels the DAFT kernel and ${\hat{x}}_{u^{★}}^{\left(B\right)}$ is a noisy copy of x that lies close to the transmit constellation, whereas any other *u* leaves a residual quadratic-phase rotation that displaces the samples from constellation points. Adopting the blind side-information detector of [17], Bob picks the candidate with the smallest decision-directed residual,

(10) $${\hat{u}}_{B}=\arg\min_{1\leq u\leq U}\mu_{u}^{\left(B\right)},\;\mu_{u}^{\left(B\right)}≜{∥{\hat{x}}_{u}^{\left(B\right)}-Q\;\left({\hat{x}}_{u}^{\left(B\right)}\right)∥}_{2}^{2},$$

where Q(·) is the nearest-neighbor slicer onto the transmit constellation. The metric $\mu_{u}^{\left(B\right)}$ is small when ${\hat{x}}_{u}^{\left(B\right)}$ concentrates near the constellation and large otherwise, so the minimizer ${\hat{u}}_{B}$ returns the hypothesis most consistent with ${\hat{s}}_{B}$. The decoded symbol vector is ${\hat{x}}_{{\hat{u}}_{B}}^{\left(B\right)}$.

Eve is modeled as a passive eavesdropper who knows the public codebook D in (3) but not the secret offset $\kappa_{AB}$. Eve therefore constructs the candidate DAFT matrices using only the public offset $\delta^{\left(u\right)}$, giving the projection

(11) $${\hat{x}}_{u}^{\left(E\right)}=E^{H}\left(c_{1},\delta^{\left(u\right)}\right)\;{\hat{s}}_{E},\;u=1,\ldots ,U,$$

and the decision

(12) $${\hat{u}}_{E}=\arg\min_{1\leq u\leq U}\mu_{u}^{\left(E\right)},\;\mu_{u}^{\left(E\right)}≜{∥{\hat{x}}_{u}^{\left(E\right)}-Q\;\left({\hat{x}}_{u}^{\left(E\right)}\right)∥}_{2}^{2}.$$

The transmitter actually used the chirp parameter $c_{2}^{\left(u^{★}\right)}=\kappa_{AB}+\delta^{\left(u^{★}\right)}$ from (2), where $\delta^{\left(u^{★}\right)}\in D$ is the public offset of the selected candidate. Eve’s hypothesis *u* therefore differs from it by $\kappa_{AB}+\delta^{\left(u^{★}\right)}-\delta^{\left(u\right)}$, which cannot be driven to zero without knowing $\kappa_{AB}$. In particular, even the correct index $u=u^{★}$ leaves the residual $\kappa_{AB}$. A quadratic-phase rotation therefore remains in ${\hat{x}}_{u}^{\left(E\right)}$ for every hypothesis, degrading both the candidate decision ${\hat{u}}_{E}$ and the symbol recovery.

The public codebook D enters the two detectors through different quantities. At Bob, the pairwise differences $\delta^{\left(u\right)}-\delta^{\left(v\right)}$ determine whether $\mu_{u}^{\left(B\right)}$ can separate two candidate projections. Two offsets too close in D produce nearly identical projections and weaken the blind decision. At Eve, the effective pairwise mismatch $\kappa_{AB}+\delta^{\left(u\right)}-\delta^{\left(v\right)}$ sets the magnitude of the residual rotation in ${\hat{x}}_{u}^{\left(E\right)}$, and a larger mismatch yields a more corrupted decision. Therefore, we are interested in studying the design of D to enlarge the mismatch at Eve while keeping the pairwise differences at Bob sufficiently large.

## 3. Security-Aware Low-PAPR Codebook Design

In this section, we first formulate the security-aware codebook design problem in Section 3.1, and then propose an alternating discrete coordinate descent algorithm to solve it in Section 3.2.

### 3.1. Secure Low-PAPR Codebook Design

Building on the observation in Section 2.2, we now quantify the pairwise differences at Bob and the pairwise mismatches at Eve through a common phase-alignment function, and formulate the codebook design as a discrete optimization problem.

Following [17], the alignment induced by a scalar chirp-parameter offset θ is measured by

(13) $$S\left(\theta \right)=\frac{1}{M}\sum_{m=0}^{M-1}\exp\;\left(j2\pi \theta m^{2}\right),$$

with |S(θ)|∈[0,1].

Note that ${\left|S\left(\theta \right)\right|}^{2}$ exhibits an irregular sidelobe pattern due to the quadratic-phase term $m^{2}$ in the DAFT kernel. Unlike the sinc-like sidelobes of linear-phase DFT systems, the peaks and nulls of ${\left|S\left(\theta \right)\right|}^{2}$ depend sensitively on θ. The codebook design exploits this non-uniform structure. Bob’s cross-alignment arguments $\delta^{\left(u\right)}-\delta^{\left(v\right)}$ must fall in low-sidelobe regions to maintain reliable detection, and Eve’s arguments $\kappa +\delta^{\left(u\right)}-\delta^{\left(v\right)}$ should also fall in low-sidelobe regions across all κ∈K to degrade Eve’s decoding. Because these two sets of arguments correspond to different θ positions, the proposed algorithm minimizes the tail-averaged metric $J_{E}\left(D\right)$ to satisfy both requirements.

A large |S(θ)| means the wrong-hypothesis projection stays near the transmit constellation and the slicer residual is small. A small |S(θ)| means the projection is pushed away from the constellation and the slicer residual is large. At Bob, since the secret offset $\kappa_{AB}$ drops out of pairwise candidate differences, the blind detector compares candidates through $\left|S\left(\delta^{\left(u\right)}-\delta^{\left(v\right)}\right)\right|$. We define Bob’s worst-case cross-alignment metric as

(14) $$\rho_{B}\left(D\right)=\max_{u\neq v}\;{\left|S\left(\delta^{\left(u\right)}-\delta^{\left(v\right)}\right)\right|}^{2},$$

which gives the least-separable candidate pair in D. A smaller $\rho_{B}\left(D\right)$ means Bob’s blind detection is more reliable. The worst-case formulation, defined as the maximum over all codeword pairs, ensures that no single codeword pair in the codebook can cause a dominant detection error for Bob. Because the blind detector in (10) selects among all *U* hypotheses, a single pair with high cross-alignment creates an error floor regardless of how well the remaining pairs are separated. This is analogous to the minimum-distance criterion in classical constellation design.

At Eve, the pairwise mismatch $\kappa_{AB}+\delta^{\left(u\right)}-\delta^{\left(v\right)}$ depends on the shared secret $\kappa_{AB}$, which is fixed in operation but unknown when D is designed. We therefore treat $\kappa_{AB}$ as an unknown parameter drawn from a finite uncertainty set K and evaluate $|S\left(\kappa +\delta^{\left(u\right)}-\delta^{\left(v\right)}\right){|}^{2}$ for every grid point κ∈K and every candidate pair u≠v. The case u=v is excluded because it reduces to ${\left|S\left(\kappa \right)\right|}^{2}$ and does not involve D. This produces a total of N=|K|U(U−1) values, which we sort in descending order as $z_{\left(1\right)}\geq z_{\left(2\right)}\geq \cdots \geq z_{\left(N\right)}$. Eve’s tail-averaged cross-alignment metric is then defined as

(15) $$J_{E}\left(D\right)=\frac{1}{K_{\beta }}\sum_{\ell =1}^{K_{\beta }}z_{\left(\ell \right)},$$

where $K_{\beta }=\left\lceil \beta N\right\rceil$ and β∈(0,1] is a fixed tail fraction. Averaging over the top $K_{\beta }$ values gives a finer discrimination among codebooks than the plain maximum $z_{\left(1\right)}$, and a smaller $J_{E}\left(D\right)$ means Eve’s largest alignments remain small across K.

For the PAPR constraint, we adopt the standard complementary cumulative distribution function (CCDF) level

(16) $$P_{q}\left(D\right)=\inf\left\{P:\mathrm{Pr}\;\left[\mathrm{PAPR}(s_{u^{★}})>P\right]\leq q\right\},$$

namely, the smallest PAPR value exceeded with probability at most *q* under the per-slot minimum-PAPR selector $u^{★}$ in (6) over D.

Based on the above metrics, the secure low-PAPR codebook design is formulated as

(17) $$\begin{array}{cc} \min_{D}\; & J_{E}\left(D\right)\\ s.t.\;\; & \rho_{B}\left(D\right)\leq \tau_{B},\\ \; & P_{q}\left(D\right)\leq \tau_{P}, \end{array}$$

where $\tau_{B}$ and $\tau_{P}$ are prescribed thresholds on $\rho_{B}\left(D\right)$ and $P_{q}\left(D\right)$, respectively, with specific values given in Section 4.

Problem (17) is non-convex and difficult to solve directly. In the next subsection, we develop an alternating discrete coordinate descent algorithm to address it.

### 3.2. Proposed Alternating Discrete Coordinate Descent Algorithm

To make Problem (17) tractable, we restrict the codebook D to a parameterized integer-grid family, reformulate the two inequality constraints as quadratic penalties, and solve the resulting discrete problem by the alternating discrete coordinate descent algorithm. Each step is detailed below.

We first restrict D to the symmetric structured family

(18) $$D\left(a\right)=\left\{\pm \frac{a_{1}}{M},\;\pm \frac{a_{2}}{M},\;\ldots ,\;\pm \frac{a_{L}}{M}\right\},\;1\leq a_{1}<a_{2}<\cdots <a_{L}\leq A_{\max},$$

parameterized by the ordered integer tuple $a=[a_{1},\ldots ,a_{L}]$ with upper bound $A_{\max}$, which gives U=2L candidates per slot. Under this parameterization, Problem (17) becomes a discrete integer program in a.

Next, we recast Problem (17) in the penalized form

(19) $$\min_{a}\;F\left(a\right)=J_{E}\left(D\left(a\right)\right)+\lambda_{B}\;{\left[\rho_{B}\left(D\left(a\right)\right)-\tau_{B}\right]}_{+}^{2}+\lambda_{P}\;{\left[P_{q}\left(D\left(a\right)\right)-\tau_{P}\right]}_{+}^{2},$$

where ${\left[x\right]}_{+}=\max\left\{x,0\right\}$ and the penalty weights $\lambda_{B},\lambda_{P}>0$ are set large enough that the minimizer of F(a) lies in the feasible region of (17).

Finally, we solve (19) by the alternating discrete coordinate descent algorithm, detailed in Algorithm 1. Starting from an initial tuple, each sweep updates the *L* coordinates of a in turn. With the convention $a_{0}≜0$ and $a_{L+1}≜A_{\max}+1$, the feasible interval for coordinate $a_{k}$ when the other coordinates are fixed is

(20) $$I_{k}\left(a\right)=\left\{a_{k-1}+1,\;a_{k-1}+2,\;\ldots ,\;a_{k+1}-1\right\},$$

which preserves the ordering in (18). Since $|I_{k}\left(a\right)|\leq A_{\max}$, we update $a_{k}$ by an exhaustive search over $I_{k}\left(a\right)$,

(21) $$a_{k}=\arg\min_{v\in I_{k}\left(a\right)}F\left(a_{1},\ldots ,a_{k-1},v,a_{k+1},\ldots ,a_{L}\right),$$

which is globally optimal with the other coordinates fixed. Sweeps continue until no coordinate reduces *F* by more than a tolerance τ.
**Algorithm 1** Proposed alternating discrete coordinate descent algorithm for problem (19)**Require:** initial tuples ${\left\{a^{\left(0,s\right)}\right\}}_{s=1}^{S}$; upper bound $A_{\max}$; penalty weights $\lambda_{B},\lambda_{P}$; tolerance τ **Ensure:** optimized tuple $a^{★}$
  1:**for **s=1,…,S** do**  2:    initialize $a=a^{\left(0,s\right)}$ and $F_{\mathrm{cur}}=F\left(a\right)$  3:    **repeat**  4:        set $F_{\mathrm{prev}}=F_{\mathrm{cur}}$  5:        **for** k=1,…,L **do**  6:            compute $I_{k}\left(a\right)$ via (20)  7:            solve $v^{★}=\arg\min_{v\in I_{k}\left(a\right)}F\left(a_{1},\ldots ,a_{k-1},v,a_{k+1},\ldots ,a_{L}\right)$  8:            compute $F_{\mathrm{try}}=F\left(a_{1},\ldots ,a_{k-1},v^{★},a_{k+1},\ldots ,a_{L}\right)$  9:            **if** $F_{\mathrm{try}}<F_{\mathrm{cur}}-\tau$ **then**10:                update $a_{k}=v^{★}$ and $F_{\mathrm{cur}}=F_{\mathrm{try}}$11:            **end if**12:        **end for**13:    **until** $F_{\mathrm{cur}}\geq F_{\mathrm{prev}}-\tau$14:    store $a^{\left(s\right)}=a$ and $F^{\left(s\right)}=F_{\mathrm{cur}}$15:**end for**16:select $s^{★}=\arg\min_{s}F^{\left(s\right)}$ and output $a^{★}=a^{\left(s^{★}\right)}$


Because F(a) is non-convex, a single descent may stop at a local minimum. We therefore run the descent from *S* initial tuples, including $a_{\mathrm{fix}}$ and several additional seed tuples, and keep the one with the lowest *F*. Each start runs at most *T* sweeps and each sweep updates *L* coordinates, yielding at most S·T·L distinct codebook evaluations in total. By comparison, the exhaustive search evaluates all AmaxL feasible codebooks. With $A_{\max}=12$, L=4, S=12, and T=4, the proposed algorithm evaluates at most 192 out of 495 codebooks and still achieves the same optimum, as confirmed in numerical results in Section 4.

### 3.3. Discussion

The secret offset $\kappa_{AB}$ and the codebook optimization serve different roles. The security gain originates entirely from $\kappa_{AB}$, which creates a de-chirping mismatch that prevents Eve from correctly recovering the transmitted symbols. The codebook optimization provides a diverse set of codewords for the per-slot selection rule in (6) to lower the transmit PAPR, while the constraint $\rho_{B}\left(D\right)\leq \tau_{B}$ ensures that Bob can reliably distinguish among them.

The proposed system operates in two phases. In the design phase, the codebook D is optimized offline before $\kappa_{AB}$ is determined. The metric $J_{E}\left(D\right)$ therefore evaluates the cross-alignment over a candidate set K that contains all possible κ values, ensuring that the designed codebook performs well regardless of which $\kappa_{AB}$ is later selected. In the operation phase, Alice and Bob first agree on a specific $\kappa_{AB}$ via an upper-layer key agreement protocol and then use the fixed codebook D for per-slot PAPR reduction. Because the key space is finite, $\kappa_{AB}$ should be periodically refreshed to prevent Eve from narrowing down its value through long-term observation.

## 4. Simulation Results and Discussions

### 4.1. Simulation Setup

In our simulations, the AFDM system parameters follow those of [17]. Each slot carries M=64 independent 16-QAM symbols with unit average power. The power amplifier follows the memoryless soft-limiter model with clipping offset $A_{\mathrm{off}}=10$ dB. Bob and Eve observe independent realizations of the doubly dispersive channel. The structured family in (18) is specialized to L=4, corresponding to U=2L=8 candidates per slot. Other key parameters are listed in Table 1.

The offset uncertainty set is K={k/M:k=±1,±2,…,±24}, which contains 48 non-zero values. We denote the codebook of [17] by $D_{\mathrm{fix}}≜D\left(a_{\mathrm{fix}}\right)$ with $a_{\mathrm{fix}}=\left[1,2,3,4\right]$. The thresholds in (17) are set to $\tau_{B}=\rho_{B}\left(D_{\mathrm{fix}}\right)$ and $\tau_{P}=P_{q}\left(D_{\mathrm{fix}}\right)+0.2$ dB, which constrain the proposed design to the separability of $D_{\mathrm{fix}}$ and to within 0.2 dB of its PAPR.

The proposed algorithm is compared with two baselines. The first is the scheme in [17], which applies the fixed codebook $D_{\mathrm{fix}}$ with $\kappa_{AB}=0$ and therefore carries no security mechanism. The second is the exhaustive-search method, which enumerates all feasible codebooks in the structured family and thus serves as the performance benchmark.

### 4.2. Simulation Results

**Figure 1:**
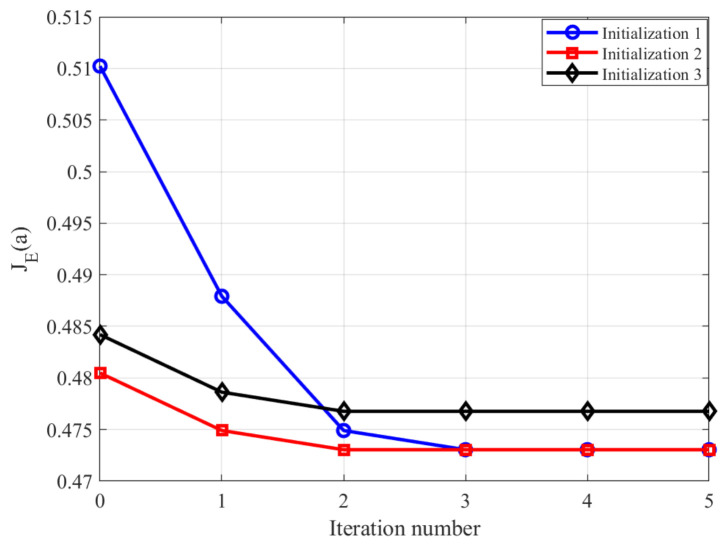
Convergence behavior of the proposed algorithm.

Figure 1 shows the convergence behavior of the proposed algorithm under three different initializations. Each curve plots the eavesdropper cross-alignment metric versus the iteration number. All three curves drop rapidly in the first two iterations and become flat after four iterations, which confirms the fast convergence and effectiveness of the proposed algorithm. This is because each coordinate update selects the optimal value over the feasible interval $I_{k}$ in (20), which guarantees non-increasing updates of the penalized objective in (19).

**Figure 2:**
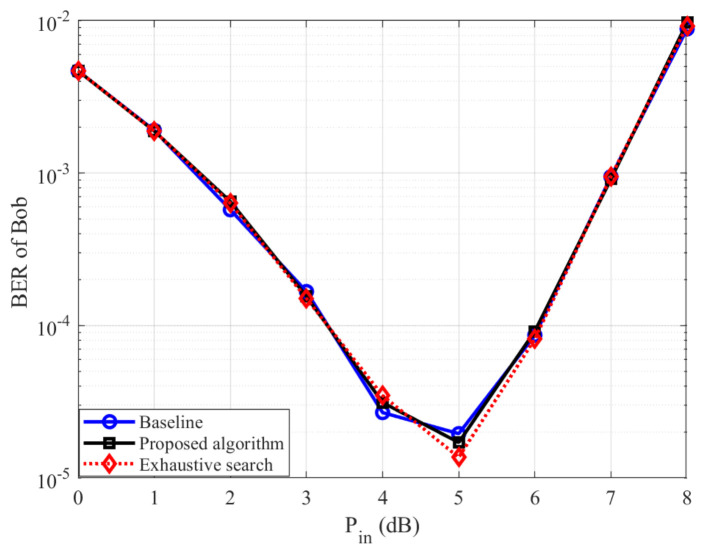
Bob BER versus transmit power under different algorithms.

**Figure 3:**
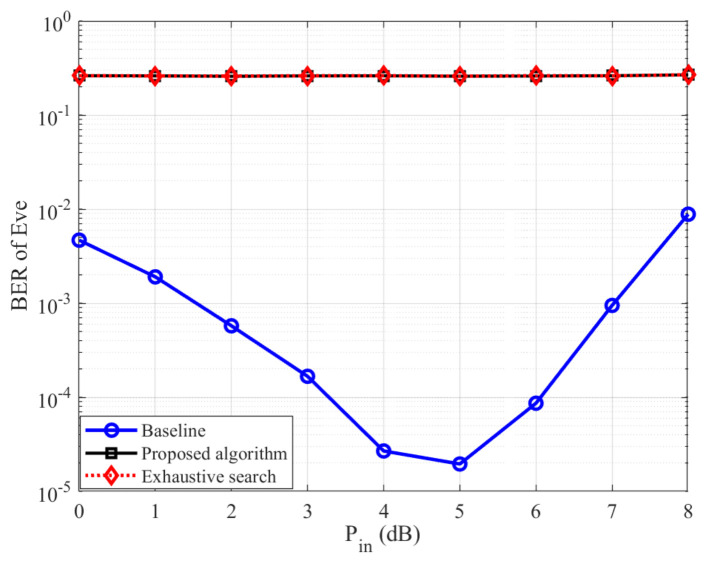
Eve BER versus transmit power under different algorithms.

Figure 2 and Figure 3 compare the BER performance of Bob and Eve, respectively, among the proposed algorithm, the exhaustive-search benchmark, and the baseline of [17], where the transmit power is swept from 0 to 8 dB. As shown in Figure 2, the three Bob BER curves essentially overlap across the full power range, and first decrease and then increase with the transmit power. The reason behind this is that the low-power region is limited by additive noise, while the high-power region is dominated by the soft-limiter clipping distortion. The overlap between the proposed algorithm and the exhaustive benchmark confirms that the codebook optimization does not degrade the decoding performance at the legitimate receiver Bob. In particular, the nearly identical Bob BER curves for the proposed and fixed codebooks at high $P_{\mathrm{in}}$ confirm that the proposed codebook is not more sensitive to clipping distortion than $D_{\mathrm{fix}}$. This is ensured by the PAPR constraint $P_{q}\left(D\right)\leq \tau_{P}$ in (19), which limits the PAPR of the optimized codebook to that of the baseline. The two codebooks therefore exhibit comparable clipping behavior at all transmit power levels.

In Figure 3, under the baseline, Eve decodes as well as Bob and follows the same BER curve, which gives the lowest Eve BER among the three schemes and therefore the weakest security. In contrast, the proposed algorithm raises Eve’s BER far above that of the baseline across the entire power range and approaches the exhaustive-search benchmark. Overall, Figure 2 and Figure 3 together show that the proposed algorithm significantly enhances the information security under the considered threat model and preserves Bob’s decoding performance. This is because the optimized codebook maintains Bob’s pairwise separability at the baseline level and drives down Eve’s strongest cross-alignment values across the entire offset uncertainty set.

**Figure 4:**
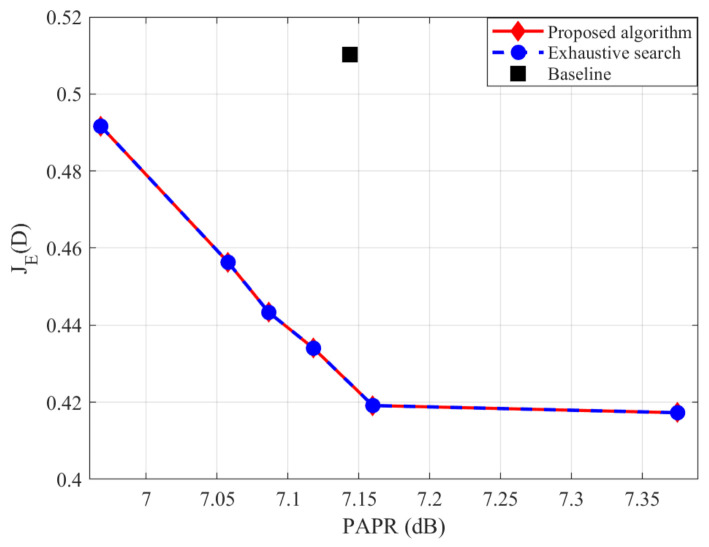
Pareto trade-off between the PAPR budget $\tau_{P}$ and the Eve cross-alignment metric $J_{E}\left(D\right)$.

Figure 4 illustrates the trade-off between the PAPR budget and the eavesdropper cross-alignment metric among the proposed algorithm, the exhaustive search, and the baseline of [17]. From Figure 4, we can see that as the PAPR budget increases, the minimum achievable cross-alignment metric decreases, which indicates the trade-off between the two metrics. Moreover, we can see that the proposed algorithm attains a substantially lower cross-alignment metric than the baseline at essentially the same PAPR level, and its curve closely approaches that of the exhaustive-search benchmark.

## 5. Conclusions

This paper proposed a security-aware codebook design for low-PAPR AFDM systems. The codebook was designed to minimize an eavesdropper-oriented cross-alignment metric subject to the legitimate user’s decoding reliability and a PAPR budget. The resulting non-convex discrete optimization problem was solved by an alternating discrete coordinate descent algorithm with quadratic-penalty reformulation and multi-start initialization. Simulation results showed that the proposed codebook preserves the legitimate receiver’s BER at the baseline level across the entire transmit power range, and the eavesdropper’s BER is significantly degraded under the considered threat model. Moreover, the proposed algorithm converges rapidly and attains the same optimum as the exhaustive search with only a fraction of the structured search space explored. 

## Figures and Tables

**Table 1:** Simulation parameters.

Parameter	Value
Number of subcarriers *M*	64
Modulation	16-QAM
Oversampling factor ζ	4
Clipping offset $A_{\mathrm{off}}$	10 dB
Positive-integer count *L*	4
Number of candidates *U*	8
Integer upper bound $A_{\max}$	12
Fixed reference $a_{\mathrm{fix}}$	[1,2,3,4]
Target CCDF level *q*	$10^{-3}$
Design-stage CCDF level	$5\times 10^{-3}$
Tail fraction β	0.10
PAPR margin	0.2 dB
Penalty weight $\lambda_{B}$	$10^{4}$
Penalty weight $\lambda_{P}$	25
Number of initial tuples *S*	12

## Data Availability

Data is contained within the article.
